# Assessing COVID-19 Mortality in Serbia’s Capital: Model-Based Analysis of Excess Deaths

**DOI:** 10.2196/56877

**Published:** 2025-04-17

**Authors:** Dane Cvijanovic, Nikola Grubor, Nina Rajovic, Mira Vucevic, Svetlana Miltenovic, Marija Laban, Tatjana Mostic, Radica Tasic, Bojana Matejic, Natasa Milic

**Affiliations:** 1 Department of Cardiovascular Diseases, University Clinical Center Zvezdara, Belgrade, Serbia; 2 Institute of Medical Statistics and Informatics, Faculty of Medicine, University of Belgrade, Dr Subotica 15, Belgrade, Serbia, 381 63367700; 3 The City Institute for Public Health, Belgrade, Serbia; 4 Clinic of Pulmonology, University Clinical Center of Serbia, Belgrade, Serbia; 5 Department of Anesthesiology, University Clinical Center of Serbia, Belgrade, Serbia; 6 The College of Health Sciences, Academy of Applied Studies Belgrade, Belgrade, Serbia; 7 Institute of Social Medicine, Faculty of Medicine, University of Belgrade, Belgrade, Serbia; 8 Division of Nephrology and Hypertension, Department of Internal Medicine, Mayo Clinic, Rochester, MN, United States

**Keywords:** COVID-19, COVID-19 impact, SARS-Cov-2, coronavirus, respiratory, infectious disease, pulmonary, pandemic, excess mortality, death rate, death toll, centralized health care, urban, Serbia, dense population, public health, surveillance

## Abstract

**Background:**

Concerns have been raised about discrepancies in COVID-19 mortality data, particularly between preliminary and final datasets of vital statistics in Serbia. In the original preliminary dataset, released daily during the ongoing pandemic, there was an underestimation of deaths in contrast to those reported in the subsequently released yearly dataset of vital statistics.

**Objective:**

This study aimed to assess the accuracy of the final mortality dataset and justify its use in further analyses. In addition, we quantified the relative impact of COVID-19 on the death rate in the Serbian capital’s population. In the process, we aimed to explore whether any evidence of cause-of-death misattribution existed in the final published datasets.

**Methods:**

Data were sourced from the electronic databases of the Statistical Office of the Republic of Serbia. The dataset included yearly recorded deaths and the causes of death of all citizens currently living in the territory of Belgrade, the capital of the Republic of Serbia, from 2015 to 2021. Standardization and modeling techniques were utilized to quantify the direct impact of COVID-19 and to estimate excess deaths. To account for year-to-year trends, we used a mixed-effects hierarchical Poisson generalized linear regression model to predict mortality for 2020 and 2021. The model was fitted to the mortality data observed from 2015 to 2019 and used to generate mortality predictions for 2020 and 2021. Actual death rates were then compared to the obtained predictions and used to generate excess mortality estimates.

**Results:**

The total number of excess deaths, calculated from model estimates, was 3175 deaths (99% CI 1715-4094) for 2020 and 8321 deaths (99% CI 6975-9197) for 2021. The ratio of estimated excess deaths to reported COVID-19 deaths was 1.07. The estimated increase in mortality during 2020 and 2021 was 12.93% (99% CI 15.74%-17.33%) and 39.32% (99% CI 35.91%-39.32%) from the expected values, respectively. Those aged 0‐19 years experienced an average decrease in mortality of 22.43% and 23.71% during 2020 and 2021, respectively. For those aged up to 39 years, there was a slight increase in mortality (4.72%) during 2020. However, in 2021, even those aged 20‐39 years had an estimated increase in mortality of 32.95%. For people aged 60‐79 years, there was an estimated increase in mortality of 16.95% and 38.50% in 2020 and 2021, respectively. For those aged >80 years, the increase was estimated at 11.50% and 34.14% in 2020 and 2021, respectively. The model-predicted deaths matched the non-COVID-19 deaths recorded in the territory of Belgrade. This concordance between the predicted and recorded non-COVID-19 deaths provides evidence that the cause-of-death misattribution did not occur in the territory of Belgrade.

**Conclusions:**

The finalized mortality dataset for Belgrade can be safely used in COVID-19 impact analysis. Belgrade experienced a significant increase in mortality during 2020 and 2021, with most of the excess mortality attributable to SARS-CoV-2. Concerns about increased mortality from causes other than COVID-19 in Belgrade seem misplaced as their impact appears negligible.

## Introduction

Discrepancies in COVID-19 mortality data for Serbia, particularly between the preliminary and final datasets of vital statistics, have raised concerns. In the original preliminary dataset, released daily during the ongoing pandemic, there was an underestimation of deaths in contrast to those reported in the subsequently released yearly vital statistics [1,2]. This finding led to the recommendation to use only the final datasets from Serbia for further research. Therefore, in this study, we aimed to validate the final mortality dataset for Belgrade, the capital of the Republic of Serbia, by collecting data on COVID-19 and non-COVID-19 causes of death and analyzing it for any evidence of cause-of-death misattribution. We utilized standardization and modeling techniques to quantify the direct impact of COVID-19 on specific age groups and to estimate subgroup-specific excess mortality. Excess mortality, defined as the increase in overall mortality rates beyond what can be anticipated by historical trends, has traditionally served as a valuable metric for estimating the mortality impact of pandemics and other extraordinary occurrences [3]. Monitoring all-cause excess deaths has been lauded as a good metric to determine the COVID-19 impact [4]. It is unaffected by the underlying clinical diagnosis and between-country differences in age and comorbidity because it facilitates comparisons between the same population [5]. While excess mortality does not equate precisely to COVID-19 fatalities, it is widely regarded as the most objective indicator available in numerous countries [3,6]. Death rate standardization and model-derived rates are explicitly compared to highlight the strengths and weaknesses of both approaches.

The literature has shown differences in the case definitions, leading to heterogeneity in COVID-19 mortality reporting and its associated metrics. Faced with the unknown threat of a growing pandemic, several approaches have emerged, ranging from counting only autopsy-confirmed cases, counting clinical diagnoses with or without laboratory confirmation, to counting all suspected cases [5,7]. The chosen approach leads to undercounting if the criteria are too stringent and overcounting if the definition is too broad. Test availability can influence the reported mortality rate if laboratory confirmation is required. In many regions, deaths attributable to COVID-19 were likely undercounted, as official reports often required a positive test result to confirm the virus as the cause of death. During the early stages of the pandemic, limited access to testing, particularly in long-term care facilities, meant that many deaths went unattributed to COVID-19. Furthermore, the systems for registering deaths vary widely between countries, affecting the consistency and comprehensiveness of reported mortality data [4,8,9]. Numerous studies have since attempted to estimate the true mortality impact of the pandemic, with the World Health Organization (WHO) estimating 3 million excess deaths globally for 2020 alone [10]. However, more detailed estimates by location remain unavailable [4,11]. It is unclear whether excess death calculations should be attempted in countries with unreliable data. Hence, focusing on settings with high-quality data may be more productive, such as cohorts with intensive data collection and accurate death ascertainment [12]. The pandemic has highlighted that death registration systems are not sufficiently robust to accommodate such large-scale events.

However, case definitions and data manipulation do not account for all differences. In addition to deaths directly caused by the SARS-CoV-2 infection, pandemic-related restrictions, such as social distancing mandates and lockdowns, likely influenced mortality rates from other causes. For example, some diseases and injuries, such as road accidents, may have decreased during the pandemic. At the same time, deaths from chronic and acute conditions might have increased due to delayed care in overwhelmed health care systems [13,14]. These shifts in mortality patterns complicate the calculation of excess deaths, as it becomes difficult to separate the impact of SARS-CoV-2 from the broader societal, economic, and behavioral changes triggered by the pandemic [8]. This challenge is particularly pronounced in countries needing more detailed cause-of-death data. While distinguishing between the myriads of influences on excess mortality would be illuminating, estimating excess mortality is a more attainable and equally important goal. Seemingly similar urban environments differed in their resilience to the COVID-19 pandemic. Certain aspects of these urban environments, such as response efficiency, optimized testing and detection strategies, and practical immunization efforts, make them more or less resilient to the spread of the SARS-CoV-2 [15]. Analyzing the impact of COVID-19 on urban environments can shed light on the epidemiological burden and help quantify the effectiveness of government policies [16]. Different geographical layouts and heterogeneous urban design have demonstrated the potential to lead to a highly nonuniform epidemic distribution [11,17]. Taking this into account, the aim of this study was to validate the final mortality dataset and assess the impact of COVID-19 on excess mortality in Belgrade, the capital of the Republic of Serbia.

## Methods

### Data Collection

The study focused on the recorded deaths and the causes of death of all citizens currently living in the territory of Belgrade for the period from 2015 to 2021. The age-specific and sex-stratified midyear population estimates for the territory of Belgrade were collected from the electronic databases of the Statistical Office of the Republic of Serbia for the given period [18]. These midyear population estimates were calculated considering the natural population change and net migration rates. Overall mortality data, as well as data on the mortality from COVID-19 as the main cause of death, were collected. The mortality data for COVID-19 as the primary cause of death were coded per the WHO guidelines [19]. These guidelines define COVID-19 as a cause of death if clinical death occurs, which is compatible with COVID-19 disease, in a case with confirmed or probable COVID-19 disease, and no alternative, more probable causes exist. For this analysis, we used the Codes for special purposes (U00-U89), Chapter XXII, *ICD-10*, where only deaths with the main cause of death–COVID-19 (U07.1, U07.2) were exclusively attributed for 2020 and 2021. This coding scheme was used to derive crude COVID-19 and non-COVID-19 mortality estimates. The data were also sourced from the electronic databases of the Statistical Office of the Republic of Serbia [18].

### Excess Death Estimation

We first calculated the standardized direct death rates for the populations from 2015 to 2019 to estimate the excess deaths [20]. The 2020 and 2021 population estimates used this standardized death rate to generate age-specific and sex-stratified mortality. This total was then compared to the actual crude death rate. Direct standardization is not without its limits. It can severely underestimate excess deaths due to COVID-19. The 5-year average estimate does not capture seasonal variation and year-to-year mortality. To account for these year-to-year trends, we use a mixed-effects hierarchical Poisson generalized linear regression model (GLM) to predict mortality for 2020 and 2021 using a yearly time series of sex, age group membership, and the logarithm of absolute population count in the region. The model was fit to mortality data observed from 2015 to 2019. To fit the Bayesian model, we estimated the parameter posterior distributions using Hamiltonian Markov Chain Monte Carlo, as implemented in Stan (version 2.26.1). The posterior distributions quantify which parameter values are most consistent with the data and are subsequently used for generating predictions. We assessed convergence by the inspection of trace plots and R-hat values, which should be below 1.01 [21], and the effective sample size, which should be greater than 1000 [22]. These quality assurance measures indicate that the sampler has achieved robust convergence and that the tails of the parameter distributions are reliable when estimating confidence intervals of excess mortality. Model out-of-sample predictive performance was assessed using leave-one-out cross-validation approximated via Pareto smoothed importance sampling [23]. Leave-one-out cross-validation metrics estimate model performance on unseen data and are useful measures of out-of-sample predictive performance. The leave-one-out cross-validation indicated that the predictors contain valuable predictive information. Priors were chosen through prior predictive simulation so that predata predictions span the range of scientifically plausible outcomes. Using weakly informative priors ensures that the model is partially regularized and protects against implausible parameter values. We constructed posterior predictive distributions to assess the model fit adequacy to evaluate predictions at the observed data’s mean (Multimedia Appendix 1) [24]. Posterior predictive distributions evaluated the models’ predictions for consistency against the observed data before any inference could be made. Furthermore, to assess model calibration across the whole observed distribution, we compared the probability integral transformed values of the predictive distribution to our data’s empirical (observed) cumulative distribution function () [25]. The model convergence diagnostic is shown in Multimedia Appendix 3. After successful model fitting, we generated predictions for each age group for the next two pandemic years and compared the expected and the observed mortalities.

The raw number of excess deaths was not comparable across countries due to significant differences in the population. To enable easier comparison across countries, we calculated the P-score. The P-Score measures the percentage difference between the reported and projected deaths.

All calculations were performed using R software (version 4.3.1; R Foundation for Statistical Computing).

### Ethical Considerations

This study was the secondary analysis of publicly available data collected by the Statistical Office of the Republic of Serbia. Data collection adhered to strict ethical guidelines ensuring the confidentiality and privacy of all participants. All collected data were fully anonymized to prevent the identification of individuals.

## Results

### Population Characteristics

From 2015 to 2019, the territory of Belgrade had an average population of 1,688,185, of which 797,080 (47.22%) citizens were male and 891,105 (52.78%) were female. The largest number of people were included in the 40‐59 year age group (459,345), closely followed by the 20‐39 year age group (459,032). The number of people aged >80 years was the lowest (72,757). These demographics remained relatively stable throughout 2021 (). The raw population characteristics are provided in .

### Death Rates

In the territory of Belgrade, there were 27,722 and 32,593 deaths in 2020 and 2021, respectively. The average number of deaths in the previous 5-year period was 23,792. In 2020 and 2021, there were 3028 (12%) and 7669 (24%) deaths when COVID-19 was coded as the primary cause.

**Figure 1.**
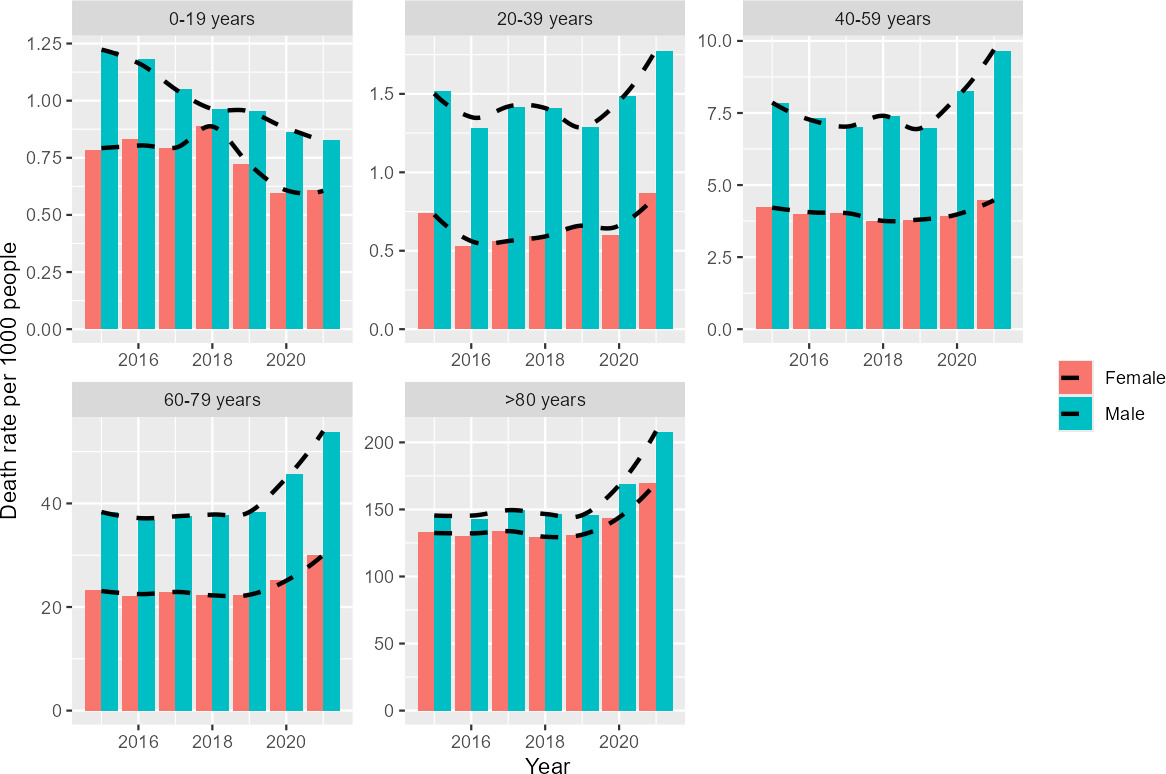
Trends of crude death rates per 1000 people stratified by sex and age group for 2015‐2021. A decrease in death rate among children and young people is evident, while all other age groups showed an increase in death rate during the pandemic (2020 and 2021) relative to the prepandemic period.

The crude death rate increased significantly in 2020 and 2021 from its average of 14.1 per 1000 people in the previous 5-year period to an average of 17.85 per 1000 people. Those in their seventh and eighth decade were the most severely impacted age groups. Age-specific crude death rates for citizens in their seventh decade of life were 33.90 per 1000 people and 40.08 per 1000 people in 2020 and 2021, respectively. Those in their eight-decade had crude death rates of 153.24 per 1000 people and 183.91 per 1000 people for 2020 and 2021, respectively. Raw death rate trends are shown in Figure 1.

### Excess Mortality

**Figure 2.**
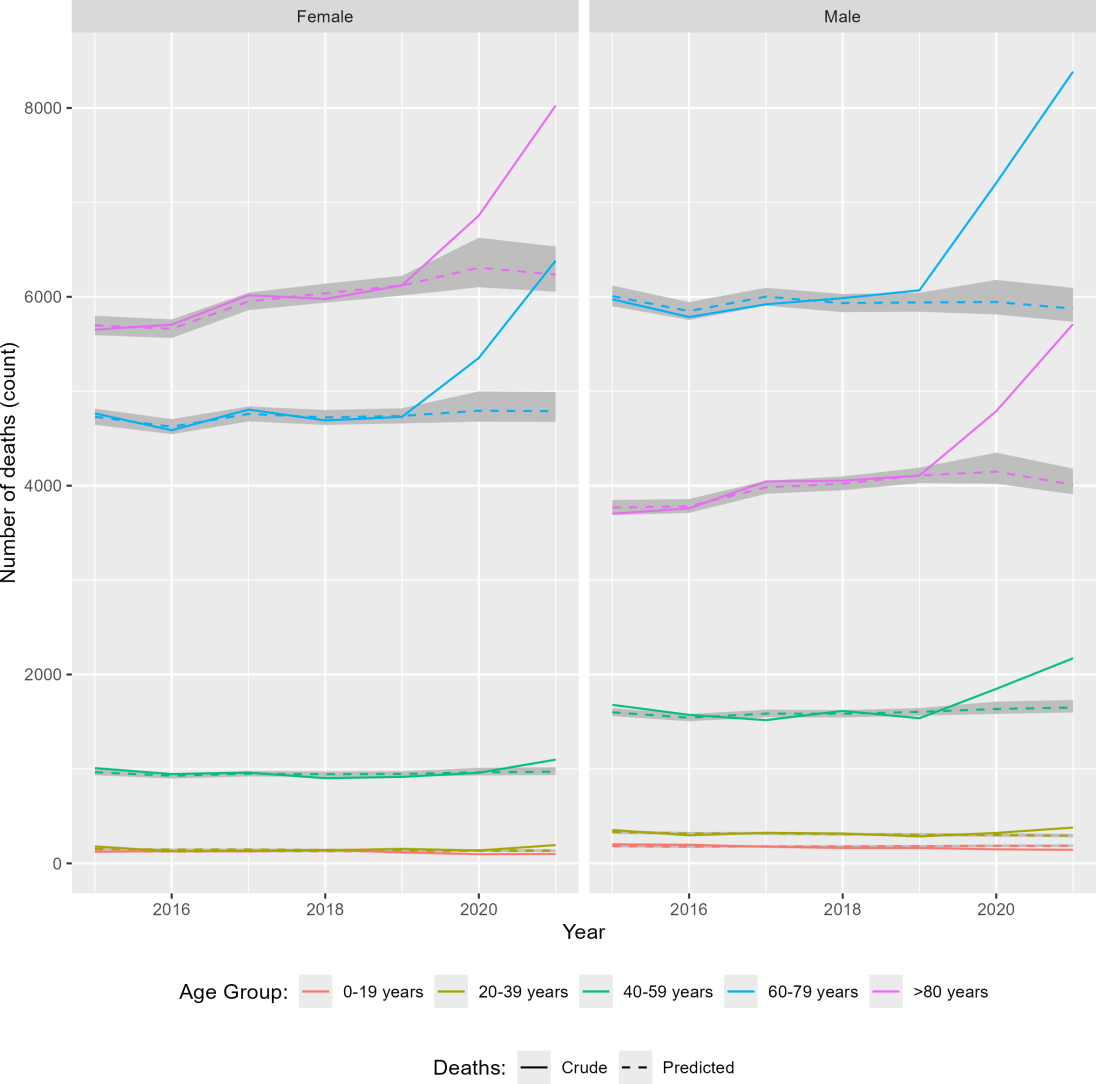
Raw death counts compared with the model-predicted death counts for the study period (2015‐2021). The raw number of deaths per year, stratified by sex and age group, is shown. Dashed lines represent model-predicted deaths. The grey-shaded area around the dashed lines represents the 99% confidence interval. An excess number of deaths is evident as a rise in the observed deaths (solid lines) compared to the prepandemic model predictions (dashed lines). A larger discrepancy between the lines indicates a larger impact of COVID-19 on those subgroups.

**Figure 3.**
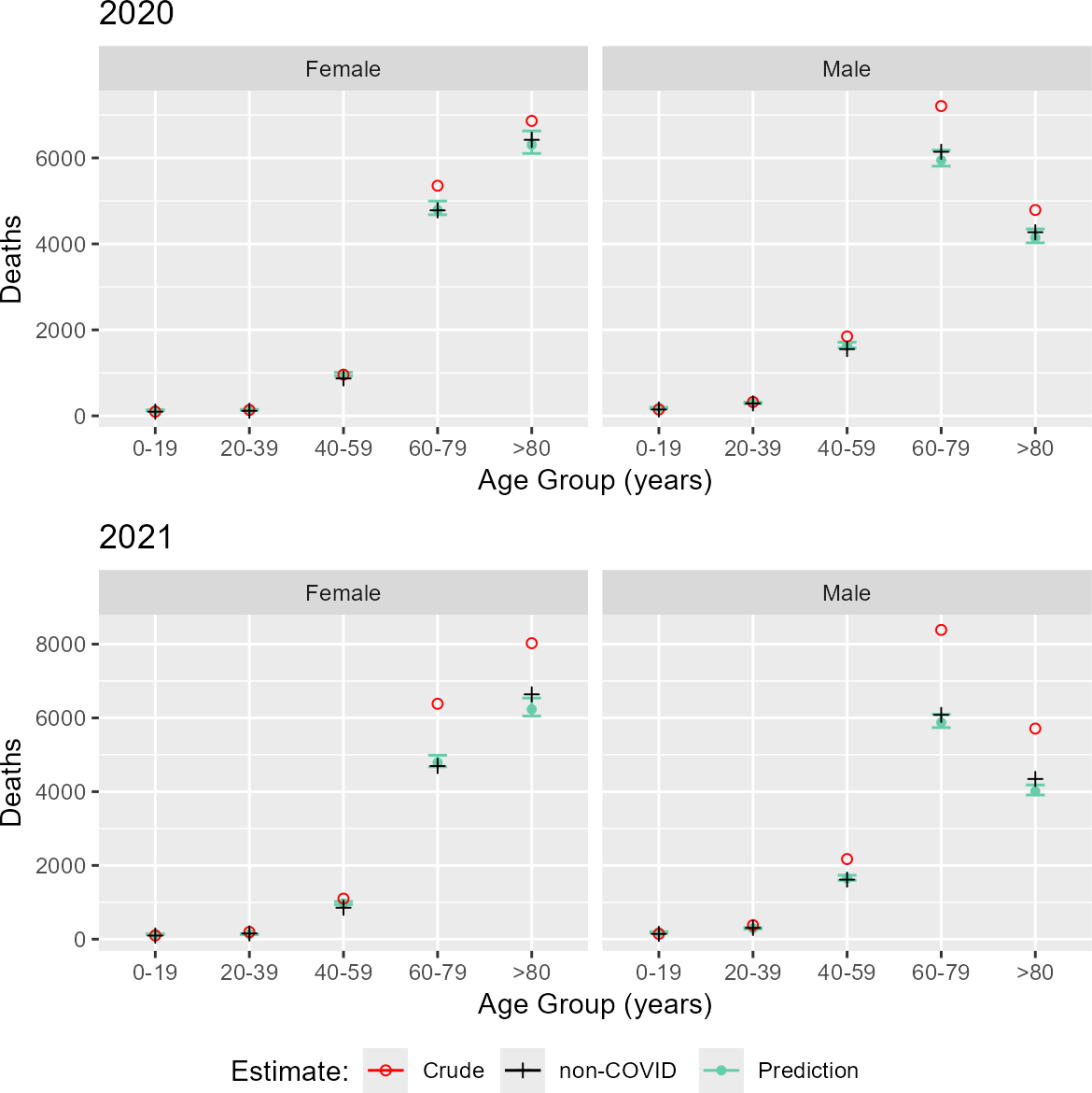
Crude, non-COVID, and model-predicted death comparisons. The model-predicted deaths fall within the confidence intervals of non-COVID deaths recorded in the territory of Belgrade. Agreement between the predicted and recorded non-COVID deaths provides evidence that cause-of-death misattribution did not occur in the territory of Belgrade for the period 2020‐2021. The error whiskers represent 99% confidence intervals of model-predicted deaths.

The total number of excess deaths, calculated from model estimates, was 3175 deaths (99% CI 1715-4094) for 2020 and 8321 deaths (99% CI 6975-9197) for 2021. The ratio of estimated excess deaths to reported COVID-19 deaths was 1.07. Excess mortality, stratified by age group and sex, is shown in Figures2^4^. The estimated increase in mortality during 2020 was 12.93% (99% CI 15.74-17.33%). During 2021, mortality increased by 39.32% (99% CI 35.91-39.32%) from the expected value. The average increase in mortality during 2020 and 2021 was 23.55%, compared to the expected mortality extrapolated from the previous 5-year period. There seemed to be fewer deaths than expected based on the predicted trends in children and young people, which aligned with the raw observed data. To be precise, between 40 and 106 and between 44 and 110 fewer deaths occurred among children and young people in 2020 and 2021, respectively; this amounted to an average decrease in mortality of 22.43% and 23.71% for this age group. The most impacted groups were people in their seventh and eighth decades. People aged 60‐79 years experienced an estimated 1279 to 2143 and 3594 to 4423 excess deaths in 2020 and 2021, respectively. People aged >80 years experienced an estimated 504 to 1625 and 2895 to 3858 excess deaths in 2020 and 2021, respectively. In people aged 60 to 79 years, there was an estimated increase in mortality of 16.95% and 38.50% in 2020 and 2021, respectively. For those aged >80 years, the increase was estimated at 11.50% and 34.14% in 2020 and 2021, respectively. For those aged up to 39 years old, there was little increase in mortality (4.72%) during 2020. However, in 2021, even those aged 20‐39 had an estimated increase in mortality of 32.95%. The crude, standardized, and model-predicted death rates are directly compared in Figure 3 and Tables1  2. The model-predicted deaths matched the number of non-COVID deaths recorded in the territory of Belgrade (Figure 3). This concordance between the predicted and recorded non-COVID deaths provided evidence that the cause-of-death misattribution did not occur in the territory of Belgrade. The error whiskers represent 99% credible intervals.

**Figure 4.**
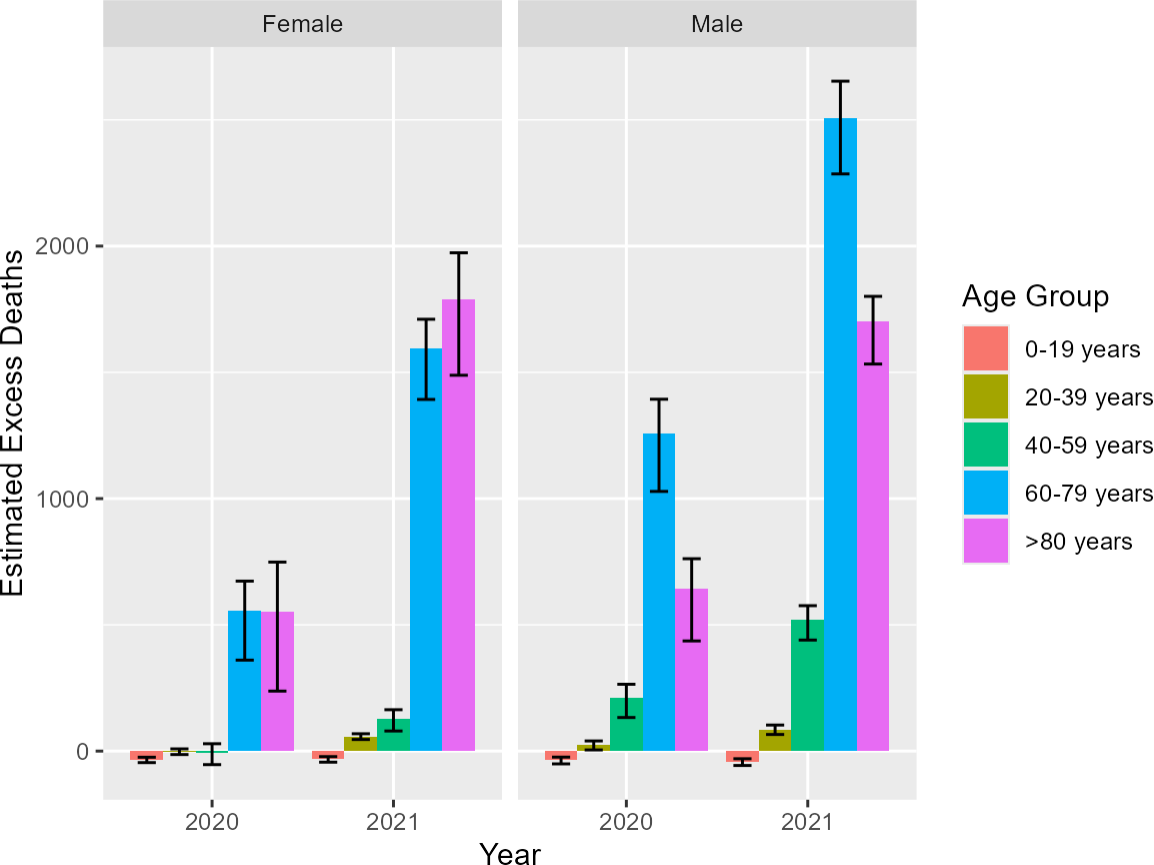
Excess deaths stratified by age group and sex during 2020 and 2021. The heights of each bar represent accumulated excess deaths for their respective year with associated 99% confidence intervals (black error whiskers). Children and young people had fewer deaths than expected, while all other age groups showed greater excess mortality. Older age groups are more severely impacted as evident by the relative height of the bars.

**Table 1.** Female-specific death rates per 1000 people.

Year	Crude death rate	Standardized death rate	Predicted death rate (99% CI)
2015	13.23	13.75	12.82‐13.54
2016	12.94	13.23	12.59‐13.27
2017	13.53	13.62	13.08‐13.74
2018	13.28	13.20	13.08‐13.78
2019	13.48	13.31	13.18‐13.86
2020	14.99	14.61	13.24‐14.66
2021	17.71	17.32	13.23‐14.56

**Table 2.** Male-specific death rates per 1000 people.

Year	Crude death rate	Standardized death rate	Predicted death rate (99% CI)
2015	15.01	15.41	14.57‐15.43
2016	14.60	14.77	14.30‐15.07
2017	15.04	15.05	14.77‐15.54
2018	15.21	15.10	14.68‐15.46
2019	15.20	15.01	14.78‐15.58
2020	17.90	17.66	14.71‐16.14
2021	21.08	21.02	14.58‐15.86

## Discussion

### Study Findings and Comparison With Previous Works

When revised death statistics were released in Serbia, some authors noted significant discrepancies between the preliminary and finalized COVID-19 dataset [1,2]. Specifically, considerable mortality underreporting occurred in the initial dataset compared to the finalized COVID-19 mortality dataset. We collected data on the number of deaths in Belgrade, coding the SARS-CoV-2 as the primary cause, to validate the accuracy of this finalized dataset. We opted for a modeling-based approach because classical standardization does not address the hypothesized problem of death misattribution, which involves coding another primary cause of death as a COVID-19 death [20]. We fitted a Poisson GLM model to the acquired data for 2015‐2019, and then used the model to generate mortality predictions for 2020 and 2021. Given the expected age and comorbidity structure within the population over the studied time frame, the model’s mortality estimates reflect the expected deaths in the absence of the pandemic. When we removed COVID-19 as a cause of death from the dataset, the model’s predictions matched the reported deaths, indicating that there were no significant cause-of-death misattributions in the final published dataset for Belgrade. Furthermore, this establishes a firm link between excess deaths and COVID-19 in Belgrade.

Possible mechanisms for this undercount discrepancy have yet to be fully investigated. Differences in the case definition were a common explanation for reporting heterogeneity between countries [5,26,27]. However, according to official reports, Serbia adopted the case definition proposed by the WHO early on during the pandemic, which remained unchanged throughout [1]. The ratio of excess mortality to the officially reported COVID-19 death count (undercount ratio) for the amended dataset was 1.07, indicating slight undercounting. This is a substantial improvement compared to the ratio of greater than 3.00 reported in the preliminary dataset [1]. The undercount ratio was larger in 2020 than in 2021, with a slight decrease in divergence. This improvement is consistent with a worldwide improvement in undercount ratios throughout the pandemic, attributed to improvements in accuracy, case definition revisions, and better reporting [3]. However, substantial undercounting remained in the preliminary dataset. Our analysis indicates that the revised datasets accurately reflect actual COVID-19 mortality, at least in Belgrade. This suggests that accurate death certificate data were collected, and that the problem was localized to the reporting system. No prior reporting system of this scale had been used in Serbia; thus, a learning curve could have existed initially, causing delays. However, the divergence between the actual and reported deaths continues into 2021, making any attribution to a learning curve unlikely.

Despite intense government measures to curb the spread of the SARS-CoV-2, the territory of Belgrade had the most considerable death rates in the country [2]. There is a complex interplay of contributing causes to this increase in mortality. Belgrade is home to one of the world’s largest medical health care centres. The University Clinical Centre of Serbia possesses a hospital floor capacity of 3150 beds [28]. The health care system in Serbia is heavily centralized, with most medical services provided by the state. Overreliance on the centralized health care system may have contributed to delays in reporting death, particularly given worldwide reports of barriers to access in rural areas [29-31]. Health care disparities between communities of differing socioeconomic status and mobility are a well-recognized moderator of pandemic impact [31-34]. The government’s response included the incorporation of many hospitals into a ‘COVID-19 Response System,’ which had limited capacity to treat non-COVID-19 diseases. Thus, treatment priorities could have contributed to limited health care access and might have increased morbidity and mortality due to other causes. However, as our model’s death predictions account for the non-COVID deaths almost perfectly, especially in the lower age subgroups, deaths from other causes could not have been acute—or at least, could not have occurred during 2020 and 2021. Increased mortality and morbidity due to the delayed diagnosis of oncological and other chronic diseases in the following years can be an expected consequence, which could manifest as a delayed increases in disease burden. Further studies in this area would shed light on the delayed impact of COVID-19.

On assessing the stringency index, a composite measure of 9 government response metrics, we found that Serbia quickly reached the maximal (strictest response) by March 22, 2020. Both stringency as well as containment and health indexes (an extension of the stringency index) settled into a 40‐70 range during 2020‐2021, briefly dropping below 40 for the period leading up to and including the June 21, 2020 elections [35]. Increased deaths led to more stringent government responses, while every reduction led to waning compliance [36]. Over time, people become less risk-averse, and government policies lose effectiveness, especially after initiating the vaccination campaign. The relative percentage of people who received all doses prescribed by the initial vaccination protocol was 46.87% by January 1, 2022, and showed significant plateauing [37]. The relative effect of nonpharmaceutical interventions in general declined by 12% from May 2021 due to less stringent methods and the introduction of vaccination strategies [38].

Mortality from COVID-19 is more significant among those with advanced age, mimicking overall mortality [39-41]. The territory of Belgrade observed a reduction in the mortality rate of children and young people (aged 0‐19 years) during 2020 and 2021. COVID-19 deaths in this age group were very low (1 case in 2020 for males and 0 for females). Most of the mortality risk in children and young people comes from injuries and infectious diseases [42]. Government intervention and the mobility restrictions during the pandemic probably contributed to lower mortality in children and young people by indirectly lowering their overall risk. For example, traffic accident fatalities decreased in the Western Balkans during lockdowns [43]. The evidence suggests a decrease in pediatric intensive care unit admissions between 2020 and 2021. Additionally, research has found no increase in adolescent self-harm or suicide [44]. These effects contributed to a decrease in the death rate among the child and adolescent population. However, the changes in health care functioning during the same period could have had long-term consequences in this age group, which have yet to manifest.

### Limitations

We acknowledge that the estimates obtained are Bayesian and are posterior distributions of expected deaths. As some of the raw non-COVID-19 deaths are on the edges of our estimates, where the predicted response parameter is least likely (given our data), there is room to argue that deaths from other causes might have increased for specific age groups. In contrast, other age groups showed fewer deaths than expected. Additionally, the resolution of our data limited our modeling choice, as the most accurate dataset only includes measurements from yearly reports.

### Conclusion

The finalized mortality dataset for Belgrade can be safely used in COVID-19 impact analysis. In 2020 and 2021, Belgrade experienced a significant increase in mortality, with the majority of the excess mortality attributable to the SARS-CoV-2. Concerns about increased mortality from causes other than COVID-19 seem misplaced, as their impact appears negligible. However, we do caution that the delayed effects of the SARS-CoV-2, barriers to health care, and the psychological impact of the COVID-19 pandemic on Belgrade territory remain unexplored.

## Supplementary material

10.2196/56877 Multimedia Appendix 1 Model posterior predictive check. Observed data (black line) compared with 100 draws from the posterior predictive distribution (light blue). Bayesian models are generative and can simulate data after successful fitting. The posterior predictive check can ensure the model has successfully learned from the data and indicates that the fit accurately predicts the mean number of expected deaths given the predictors.

10.2196/56877 Multimedia Appendix 2 Model probability integral transform (PIT) values plotted against the empirical cumulative distribution function (ECDF). The curves fall across the diagonal and stay within the confidence bands, indicating that the model’s predictive distribution is well-calibrated. This ensures that the predictions are reliable across the whole range of the distribution and that the model is not under- or overestimating deaths for certain subgroups.

10.2196/56877 Multimedia Appendix 3 Model diagnostic Markov Chain Monte Carlo (MCMC) chain trace plots show adequate chain mixing for estimated parameters. MCMC chains are used to sample parameter values from the model and their adequate mixing ensures that the predictions are stable and consistent with the observed data. The chains ran for 4000 iterations, with an adaptive warm-up period of 1000 iterations.

10.2196/56877 Multimedia Appendix 4 Demographic pyramid comparison between the years 2015 and 2021. Relative age and sex subgroups remained stable over the study period ensuring that the baseline populations are comparable.

10.2196/56877 Multimedia Appendix 5 Midyear serial baseline population structure in Belgrade, Serbia’s capital stratified by age and sex. Midyear population estimates were calculated considering natural population change and net migration rates.

## Abbreviations

GLM: generalized linear regression model
WHO: World Health Organization

## References

[1] Galjak M, Marinković I (2023). Discrepancies between preliminary and final COVID-19 mortality data-the case of Serbia. Ann Epidemiol.

[2] Marinkovic I, Galjak M (2021). Excess mortality in Europe and Serbia during the COVID-19 pandemic in 2020. Stanovnishtvo.

[3] Karlinsky A, Kobak D (2021). Tracking excess mortality across countries during the COVID-19 pandemic with the World Mortality Dataset. Elife.

[4] COVID-19 Excess Mortality Collaborators (2022). Estimating excess mortality due to the COVID-19 pandemic: a systematic analysis of COVID-19-related mortality, 2020-21. Lancet.

[5] Corrao G, Rea F, Blangiardo GC (2021). Lessons from COVID-19 mortality data across countries. J Hypertens.

[6] Beaney T, Clarke JM, Jain V (2020). Excess mortality: the gold standard in measuring the impact of COVID-19 worldwide?. J R Soc Med.

[7] Feyissa GT, Tolu LB, Ezeh A (2021). COVID-19 death reporting inconsistencies and working lessons for low- and middle-income countries: opinion. Front Med (Lausanne).

[8] Lau H, Khosrawipour T, Kocbach P, Ichii H, Bania J, Khosrawipour V (2021). Evaluating the massive underreporting and undertesting of COVID-19 cases in multiple global epicenters. Pulmonol.

[9] Kobak D (2021). Excess mortality reveals Covid’s true toll in Russia. Signif (Oxf).

[10] World Health Organization. The true death toll of COVID-19: estimating global excess mortality.

[11] GBD 2021 Demographics Collaborators (2024). Global age-sex-specific mortality, life expectancy, and population estimates in 204 countries and territories and 811 subnational locations, 1950-2021, and the impact of the COVID-19 pandemic: a comprehensive demographic analysis for the Global Burden of Disease Study 2021. Lancet.

[12] Ioannidis JPA, Zonta F, Levitt M (2023). Flaws and uncertainties in pandemic global excess death calculations. Eur J Clin Invest.

[13] Mungmunpuntipantip R, Wiwanitkit V (2021). The COVID-19 pandemic and traffic accidents. S Afr Med J.

[14] Salottolo K, Caiafa R, Mueller J (2021). Multicenter study of US trauma centers examining the effect of the COVID-19 pandemic on injury causes, diagnoses and procedures. Trauma Surg Acute Care Open.

[15] Truszkowska A, Fayed M, Wei S (2022). Urban determinants of COVID-19 spread: a comparative study across three cities in New York state. J Urban Health.

[16] Salama AM (2020). Coronavirus questions that will not go away: interrogating urban and socio-spatial implications of COVID-19 measures. Emerald Open Res.

[17] Brizuela NG, García-Chan N, Gutiérrez Pulido H, Chowell G (2021). Understanding the role of urban design in disease spreading. Proc R Soc A.

[18] Republika srbija [Article in Serbian]. Republički zavod za statistiku.

[19] World Health Organization. International Guidelines for Certification and Classification (Coding) of COVID-19 as Cause of Death.

[20] Naing NN (2000). Easy way to learn standardization: direct and indirect methods. Malays J Med Sci.

[21] Vehtari A, Gelman A, Simpson D, Carpenter B, Bürkner PC (2021). Rank-normalization, folding, and localization: an improved Rˆ for assessing convergence of MCMC (with discussion). Bayesian Anal.

[22] Bürkner PC (2017). brms: An R package for Bayesian multilevel models using Stan. J Stat Soft.

[23] Vehtari A, Simpson D, Gelman A, Yao Y, Gabry J (2022). Pareto smoothed importance sampling. arXiv.

[24] Gabry J, Simpson D, Vehtari A, Betancourt M, Gelman A (2019). Visualization in Bayesian workflow. J R Stat Soc Ser A Stat Soc.

[25] Säilynoja T, Bürkner PC, Vehtari A (2022). Graphical test for discrete uniformity and its applications in goodness-of-fit evaluation and multiple sample comparison. Stat Comput.

[26] Marinković I, Tramošljanin A, Galjak M (2023). Assessing the availability and quality of COVID-19 mortality data in Europe: a comparative analysis. Eur J Public Health.

[27] Seghezzo G, Allen H, Griffiths C (2024). Comparison of two COVID-19 mortality measures used during the pandemic response in England. Int J Epidemiol.

[28] Uredba o planu mreže zdravstvenih ustanova [Article in Serbian]. https://www.paragraf.rs/propisi/uredba_o_planu_mreze_zdravstvenih_ustanova.html.

[29] Azar KMJ, Shen Z, Romanelli RJ (2020). Disparities in outcomes among COVID-19 patients in a large health care system in California. Health Aff (Millwood).

[30] Núñez A, Sreeganga SD, Ramaprasad A (2021). Access to healthcare during COVID-19. Int J Environ Res Public Health.

[31] Abedi V, Olulana O, Avula V (2021). Racial, economic, and health inequality and COVID-19 infection in the United States. J Racial Ethn Health Disparities.

[32] Bambra C, Riordan R, Ford J, Matthews F (2020). The COVID-19 pandemic and health inequalities. J Epidemiol Community Health.

[33] Solís Arce JS, Warren SS, Meriggi NF (2021). COVID-19 vaccine acceptance and hesitancy in low- and middle-income countries. N Med.

[34] Dror AA, Eisenbach N, Taiber S (2020). Vaccine hesitancy: the next challenge in the fight against COVID-19. Eur J Epidemiol.

[35] Hale T, Angrist N, Goldszmidt R (2021). A global panel database of pandemic policies (Oxford COVID-19 Government Response Tracker). Nat Hum Behav.

[36] Agyapon-Ntra K, McSharry PE (2023). A global analysis of the effectiveness of policy responses to COVID-19. Sci Rep.

[37] Mathieu E, Ritchie H, Rodés-Guirao L (2020). Coronavirus Pandemic (COVID-19).

[38] Ge Y, Zhang WB, Wu X (2022). Untangling the changing impact of non-pharmaceutical interventions and vaccination on European COVID-19 trajectories. Nat Commun.

[39] Goldstein JR, Lee RD (2020). Demographic perspectives on the mortality of COVID-19 and other epidemics. Proc Natl Acad Sci U S A.

[40] Shang W, Wang Y, Yuan J, Guo Z, Liu J, Liu M (2022). Global excess mortality during COVID-19 pandemic: a systematic review and meta-analysis. Vaccines.

[41] Safavi-Naini SAA, Farsi Y, Alali WQ, Solhpour A, Pourhoseingholi MA (2022). Excess all-cause mortality and COVID-19 reported fatality in Iran (April 2013-September 2021): age and sex disaggregated time series analysis. BMC Res Notes.

[42] Flaxman S, Whittaker C, Semenova E (2023). Assessment of COVID-19 as the underlying cause of death among children and young people aged 0 to 19 years in the US. JAMA Netw Open.

[43] Transport Community (2021). Fatalities for 2020 annual statistics for Western Balkans. https://www.transport-community.org/publications/fatalities-for-2020-annual-statistics-for-western-balkans-2/.

[44] McCluskey CK, Black TR, Zee-Cheng J (2024). Suicide and self-harm in adolescents during the COVID-19 pandemic: A U.S. virtual pediatric systems, LLC, database study of PICU admissions, 2016-2021. Pediatr Crit Care Med.

[45] Statistical Office of the Republic of Serbia. Deaths by causes of death, sex and age.

[46] Statistical Office of the Republic of Serbia. Estimates of population by age and sex (beginning, middle and end of year).

