# Supplementary figures and images for: Assessing COVID-19 Mortality in Serbia’s Capital: Model-Based Analysis of Excess Deaths
Source: JMIR Public Health Surveill. 2025 Apr 17;11:e56877. doi: 10.2196/56877 (PMC12021472; doi:10.2196/56877)

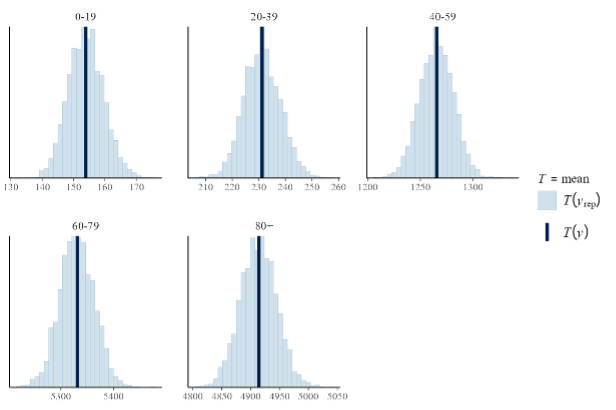

Supplement: Multimedia Appendix 1 [file publichealth-v11-e56877-s001.png]

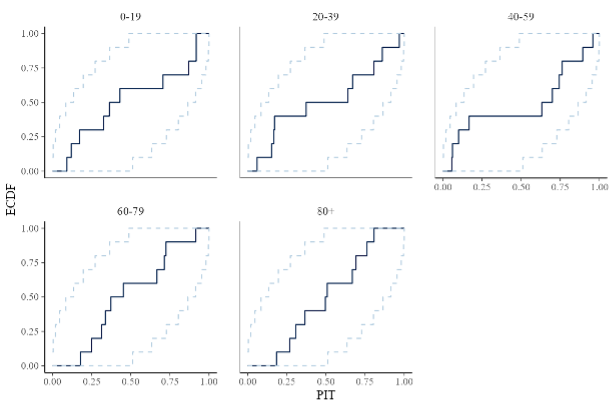

Supplement: Multimedia Appendix 2 [file publichealth-v11-e56877-s002.png]

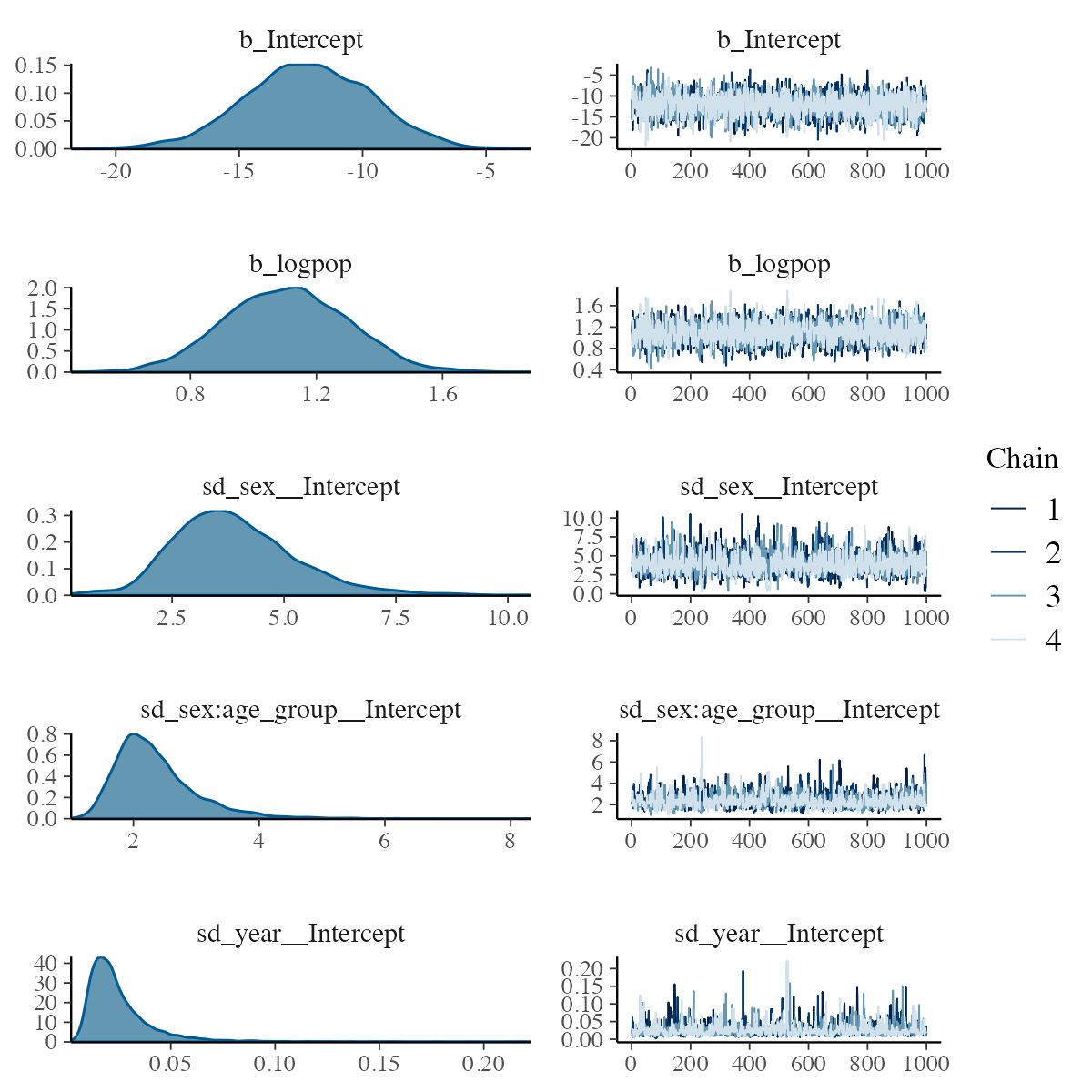

Supplement: Multimedia Appendix 3 [file publichealth-v11-e56877-s003.png]

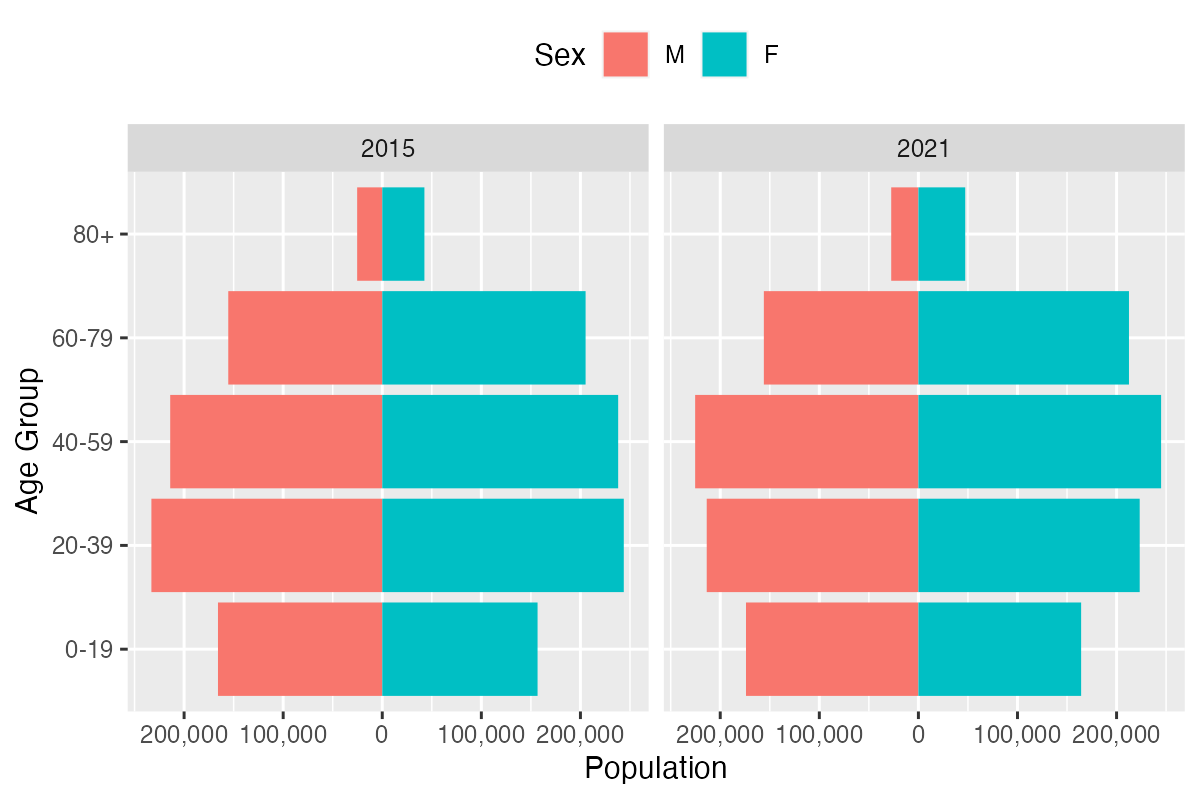

Supplement: Multimedia Appendix 4 [file publichealth-v11-e56877-s004.png]
